# Experiences with HPTN 067/ADAPT Study-Provided Open-Label PrEP Among Women in Cape Town: Facilitators and Barriers Within a Mutuality Framework

**DOI:** 10.1007/s10461-016-1458-y

**Published:** 2016-06-17

**Authors:** K. Rivet Amico, Melissa Wallace, Linda-Gail Bekker, Surita Roux, Millicent Atujuna, Elaine Sebastian, Bonnie J. Dye, Vanessa Elharrar, Robert M. Grant

**Affiliations:** 1 Department of Health Behavior and Health Education, University of Michigan, Ann Arbor, MI USA; 2 The Desmond Tutu HIV Centre, Cape Town, South Africa; 3 Dept of Medicine and Institute of Infectious Disease and Molecular Medicine, The Desmond Tutu HIV Centre, Cape Town, South Africa; 4 FHI 360, Durham, NC USA; 5 Clinical Prevention Research Branch, PSP/DAIDS/NIAID/NIH, Bethesda, MD USA; 6 Gladstone Institutes, San Francisco AIDS Foundation, University of California, San Francisco, CA USA

**Keywords:** PrEP, Open-label, Women, Adherence, Mutuality framework, Barriers, South Africa

## Abstract

Placebo-controlled trials of pre-exposure prophylaxis (PrEP) have reported challenges with study-product uptake and use, with the greatest challenges reported in studies with young women in sub-Saharan Africa. We conducted a qualitative sub-study to explore experiences with open-label PrEP among young women in Cape Town, South Africa participating in HTPN 067/Alternative Dosing to Augment Pre-Exposure Prophylaxis Pill Taking (ADAPT). HPTN 067/ADAPT provided open label oral FTC/TDF PrEP to young women in Cape Town, South Africa who were randomized to daily and non-daily PrEP regimens. Following completion of study participation, women were invited into a qualitative sub-study including focus groups and in-depth interviews. Interviews and groups followed a semi-structured guide, were recorded, transcribed, and translated to English from isiXhosa, and coded using framework analysis. Sixty of the 179 women enrolled in HPTN 067/ADAPT participated in either a focus group (six groups for a total of 42 participants) or an in-depth interview (n = 18). This sample of mostly young, unmarried women identified facilitators of and barriers to PrEP use, as well as factors influencing study participation. Cross-cutting themes characterizing discourse suggested that women placed high value on contributing to the well-being of one’s community (Ubuntu), experienced a degree of skepticism towards PrEP and the study more generally, and reported a wide range of approaches towards PrEP (ranging from active avoidance to high levels of persistence and adherence). A Mutuality Framework is proposed that identifies four dynamics (distrust, uncertainty, alignment, and mutuality) that represent distinct interactions between self, community and study and serve to contextualize women’s experiences. Implications for better understanding PrEP use, and non-use, and intervention opportunities are discussed. In this sample of women, PrEP use in the context of an open-label research trial was heavily influenced by underlying beliefs about safety, reciprocity of contributions to community, and trust in transparency and integrity of the research. Greater attention to factors positioning women in the different dynamics of the proposed Mutuality Framework could direct intervention approaches in clinical trials, as well as open-label PrEP scale-up.

## Introduction

HIV prevention science has produced considerable advances in recent years, with clinical trials demonstrating effectiveness of oral pre-exposure prophylaxis (PrEP) for prevention of HIV transmission [1–4]. Results from the iPrEx randomized controlled trial (RCT) [3], Partners PrEP study [1], and CDC’s TDF2 study [4] led to US Food and Drug Administration approval of an indication for the first publically available medication to prevent HIV [5]. Recent findings from IPERGAY [6] and PROUD [7], both involving PrEP in cohorts of men who have sex with men (MSM) in France and Canada and the UK, respectively, provide additional support for the effectiveness of PrEP. In contrast, results from the FEM-PrEP study [8] and VOICE [9], both involving women in sub-Saharan Africa, did not demonstrate effectiveness of oral PrEP in the setting of very low rates of PrEP use (cf., [10]). Varying rates of adherence to study product has become a well-recognized threat in PrEP trials and projects [11].

In the wake of the low study-product use observed in studies with sub-Saharan African women, concerns have been raised about the overall acceptability and feasibility of oral PrEP regimens in this population. However, recent qualitative explorations of factors that may have influenced product use in these trials [12–15] suggest that aspects of being in a trial testing an investigational biomedical agent may have heavily influenced product non-use. Feelings towards the research, such as overall support for the project’s goals [12] or alternatively ambivalence towards it [15] influenced use and non-use. It is possible that issues inherent in clinical trials with investigational drugs may not generalize to acceptability or feasibility of open-label PrEP. To date, however, experiences among women in sub-Saharan African women with open-label PrEP, specifically, have not been characterized.

The HPTN 067/ADAPT trial was one of the first open label PrEP studies to be conducted with women in sub-Saharan Africa [16]. This Phase II, randomized, open-label clinical trial of oral emtricitabine/tenofovir disoproxil fumarate (FTC/TDF) PrEP investigated whether non-daily versus daily regimens resulted in equivalent prophylactic pre- and post-sex coverage. After 6 weeks of once a week directly observed dosing, participants were randomly assigned to one of three unblinded PrEP dosing regimens for 24 weeks of self-administration: daily, twice weekly with a post-sex dose, or event-driven with before and after sex dosing. Only the daily regimen was known to be effective while the study was conducted, and the other two non-daily regimens were presented to participants as investigational.

The aim of HPTN 067/ADAPT was presented to participants as focused on participants’ real-world experiences with trying to follow their assigned regimen. Non-adherence was framed as an important and understandable experience and participants were supported with education around their assigned regimen, skills building for dosing-schedules and forecasting of sexual events, and motivational support through Next Step Counseling [17] at each study visit. Pills were dispensed from a Wisepill™ device that recorded each opening and weekly interviews with interviewers tasked solely with collecting pill-taking and sex event data without feedback to study counsellors or clinicians were conducted.

Three sites took part in HPTN 067/ADAPT, including a site in Cape Town, South Africa enrolling heterosexual women, and sites in Bangkok, Thailand and New York City, USA, enrolling men who have sex with men and transgender women. To explore South African women’s experiences with open-label PrEP provided within the context of a research study, we conducted a qualitative sub-study with women participating in HPTN 067/ADAPT at the Cape Town site. The aim of the qualitative sub study was to provide a nuanced understanding of PrEP use in the context of individual, community, study, and product dynamics. Specifically, we sought to identify global and regimen specific facilitators and barriers to study-provided PrEP use and participation in the open-label PrEP study, and the overarching cross-cutting themes in the narratives that contextualized women’s experiences. Similar to previous work in this area [14], we approached the data with the assumption that the levels identified in the socioecological model would generally organize emerging themes. We sought to extend our understanding of these factors by proposing how these levels interact to explain various approaches participants had towards study-provided PrEP across the full range of use and non-use, which reflected initiation or uptake (or avoidance of it), persistence (essentially, adoption of the regimen as something the participant is trying to do), and adherence execution (extent to which the participant is following the dose requirements of the regimen). These approaches to PrEP use emanated from discourse in the current study, but is generally comparable to a recently proposed typology generated from qualitative work in the VOICE study, where patterns of study-product use included non-initiation, discontinuation, mis-implementation, and adherence [18].

In addition to presenting participant reported facilitators and barriers, we identify overarching cross-cutting themes that appeared to characterize aspects of participation and PrEP use that were particularly influential to participant experiences. Drawing from these results, related literature [13–15, 19], and well-vetted social behavioral (socio-ecological [20] and attitude formation [21]) and community (community based participatory research [22]) models, we propose an organizing framework (a Mutuality Framework) to explain different participant approaches to study-provided PrEP. We propose that a participant’s approach to study-provided PrEP is the direct result of a specific, definable interplay between participant, study, and community factors, which depend heavily on one’s sense of trust in PrEP. The framework encompasses four dynamics labeled by the dominating type of relationship women in that dynamic are anticipated to have with PrEP and the institutions providing it (in the current case, the study): (1) distrust, (2) uncertainty, (3) alignment and (4) mutuality. Intervention strategies targeting enhancing alignment (i.e., positive beliefs in PrEP and goals of the research project) and mutuality (i.e., sense of ownership over and advocacy towards PrEP and goals of the research project) are suggested.

## Methods

From the 179 women randomised to one of the three regimens in the parent study, we planned to recruit a total of 60 participants (34 % of total sample) for participation in either focus group (FG) discussions or in-depth interviews (IDIs), with a similar interview-guide used for FG and IDIs. Per arm, two FGs and six IDIs were planned (for a total of six FGs and 18 IDIs). Sample size was advised by ‘information power’ [23], where our targeted recruitment reflected the discrete aims of the study and the specificity in inquiry (e.g., use of the same basic semi-structured guide in all FGs and IDIs) and participant groups (e.g., all attended the same clinic, interacted with product, and resided in the same general community). Inclusion criteria for the qualitative sub-study was having finished the on-drug portion of the study (study week 34), while exclusion was having finished over 3-months ago. Convenience sampling was used to identify FG participants, where eligible women were informed of open spots in planned FGs and referred to a coordinator if interested. IDI participants were identified with a combination of convenience and targeted sampling, where attempts were made to include at least two participants from each arm who may have had low adherence based on staff impressions (not Wisepill™ or drug level testing as that data was not yet available at time of recruitment). Similar to FG recruitment, women were informed of open spots for interviews and scheduled if interested.

FGs and IDIs were conducted in participant preferred language (isiXhosa or English) by an independent experienced interviewer who was not part of the clinical study team and had several years of experience in conducting qualitative interviews with women in the communities surrounding the research site. The development of the interview guide and the approach to analysis of data was situated within a socio-ecological framework [20] and aspects of the information, motivation and behavioral skills model [24] adapted to the current context. The main areas of inquiry from the interview-guide are presented in Table 1.

**Table 1:** Interview guide areas of inquiry for focus groups (FGs) and in-depth-interviews (IDIs)

Domain	Inquiries/prompts
Feasibility/acceptability	Perceptions of feasibility, acceptability and ease of uptake for their assigned regimen
Alteration of regimen	Altering the regimen to better ‘fit’ their daily life or risk behavior
Preference for other regimens	Whether participant(s) would switch to a different regimen if available; what the ideal regimen would be
Facilitators and barriers to adherence	Common facilitators and barriers to following assigned regimen
Disclosure of participation	Sense of importance that others knew the participant was enrolled in the study
Experiences with participation and study team	Feelings towards participation, the project, project-staff, and how pill-taking and condom use was supported
Recommendations	Recommendations for change in study support or adherence support approach

Data was transcribed and translated into English and analysed using a thematic framework analysis approach [25–27]. Two trained coders sorted transcribed discourse into “frames” determined by interviewer inquiry (which was based on the semi-structured guide). Each frame was then iteratively reviewed for main themes in participant responses to interviewer inquiries. The coding team met throughout this process to review and refine themes, with any disagreements resolved through discussion. Methods were less to ensure interrater reliability [28] than to promote adaptions in the code book that leverage unique insights of coders to create a common, nuanced understanding of frames and themes, which has been evaluated as an approach that produces high level of agreement [29]. Final codes and themes had consensus between coders. Themes in each frame and example quotes supporting the themes were identified, followed by a review and synthesis of all framed content spanning across multiple areas to identify individual, local, cultural or group beliefs and experiences that contextualized women’s experiences in the study. Important observations from the qualitative data that were not well captured in the thematic coding from the framework analyses were also evaluated for potential inclusion in cross-cutting themes. These cross-cutting themes advised our formation of a Mutuality Framework that sought to explain women’s different approaches to study-provided PrEP on the basis of interactions between the participant, the study and study-provided PrEP, and the community. Model development was led by the lead author with iterative vetting with the coding team and the site’s community liaisons.

## Results

### Participants

As planned, 60 women participated in the sub-study (42 in focus groups and 18 in interviews). Women were 18–44 years of age (average 26, SD 7), with the majority under the age of 25. The vast majority (90 %) were not married. Both younger age and being unmarried distinguished the sub-study participants from the study cohort, however the groups were comparable on other demographic or sexual behaviour data.

### Themes

#### Facilitators of and Barriers to Study-Provided PrEP Use

As detailed in Table 2, several themes emerged in discourse surrounding facilitators of adherence to the study-provided PrEP pills. This content was organized into the following themes: (1) Efficacy beliefs in PrEP providing effective protection against HIV, (2) perceived HIV-prevention needs/risks highlighted in discourse around enhanced sense of vulnerability to HIV and identifying PrEP use as a source of protection in the event of rape or forced sex, (3) use of concrete adherence strategies such as reminders or pocket dosing, and (4) social support from important significant others that provided concrete help with dose-taking and also removed study-participation disclosure-related barriers. Barriers to adherence, presented in Table 2, included; (1) Attributes of the PrEP pills (e.g., taste and smell) that made dosing unpleasant, (2) perceived side-effects reported largely as nausea and headaches either experienced directly or indirectly through reports of other participants, (3) ARV-related stigma associated with others assuming the participant is/was HIV-positive because of being seen taking “HIV-medications”, and (4) needs for privacy or non-disclosure to important others making dosing more difficult or not possible without risk of undesired disclosure of being part of the study. Specific to non-daily arms, discussion on sex-dependent dosing revealed challenges in predicting sex for pre-sex dosing, but largely centred on difficulty with post-sex dosing because of a perceived mis-match between relaxation or rest following sex and the action-oriented steps needed to take a post-sex dose. Themes and example quotes in Table 2 highlight experiences intentionally limited to facilitators and challenges discussed in relation to dose taking (i.e., regimen execution); other factors that influenced multiple aspects of participation in the study are presented separately below in themes for study participation and engagement more generally. Of note, several of the factors eroding participation in the study noted below have clear implications for also creating challenges to adherence.

**Table 2:** Facilitators of and barriers to study-provided PrEP use

Theme	Defined as discourse on…	Example quotes
Facilitators of PrEP use		
Efficacy beliefs	Beliefs that PrEP works to prevent HIV	‘What motivated me is the fact that they protect me from getting HIV, because sometimes I forget to use a condom with my boyfriend that is why I continued using the pills. I had that hope that the pills will protect me…” D IDI “The treatment made me safe so I continued taking the pills.” E IDI “I heard here at the site that these pills work and that they were being tested overseas too and that the results proved that these pills do work so that made me take the pills.” E FG
Perceived HIV-prevention needs/risks	Risk of being exposed to HIV/desire to protect HIV-negative status; discussion of prevention in context of rape/forced sex	“As I said before, it made me want to protect myself. Before I was involved in the study, I didn’t care as much as I do now.” T IDI “… I also knew that this pill will help me in any case like if I was to be raped I would not be infected with HIV” D IDI
Use of concrete adherence strategies	Strategies used for adherence	“I didn’t set my phone or anything like that. I knew that if Generations [a popular television series] is about to begin, I would take my pill.” D FG “I would keep the tablets in my pocket so that I always remember to drink the tablets” E IDI
Social support for use	Support from partner/friend/family for taking PrEP	“My friends would also help me because they knew at a certain time I was supposed to take the pill. So it was those kinds of things that helped me.” T FG “The boyfriend that I was staying with was very supportive and he always encourages me to drink the tablets.” E IDI
Barriers to PrEP use		
Attributes of PrEP pills (taste, smell)	Negative perceptions of pill attributes	“Yes, at the beginning I was asking myself, how am I going to be able to swallow this big pill and as time goes on, I was able to swallow them.” D IDI “What I found difficult was the way it smelled, it made me nauseous. So when the time came for me to take it, I had to think hard about it. I wasn’t too happy taking it.” D FG
Side-effects attributed to PrEP	Negative physical experiences attributed to using PrEP in self or others	“At first it was hard because they were not good for my immune system but they have told me here that at first I might have some side effects such as always feel[ing] hungry, dizziness and they made me to have a small rush but as time goes on, I got used to it.” D IDI
ARV-related stigma	Fears that PrEP use will be misattributed to HIV-treatment; participant will be assumed to be HIV-positive	“Plus negative response from friends … they compare Truvada^®^ to ARVs because they know someone who was taking the same medication and ended up being HIV positive.” D IDI “We are very shy of walking around with pills in our bags, because we are scared of what people would say, because let us say you take out your pills and take them at the party, some people won’t even ask- they will just say it’s an ARV.” E FG
Needs for privacy/non-disclosure	Non-disclosure of study participation to significant others, due to anticipated stigma, misunderstanding or lack of support	“The problem was that I didn’t tell my boyfriend that I was taking the Truvada^®^. So when I went to his place, I wouldn’t take it along” T FG “So you are now sitting with friends and you see that the time is about to arrive. So what will they say if I were to take these pills in front of them? My friends are going to judge me. So I end up not taking them then.” E FG
Non-daily regimens		
Sex-dependent doses	Difficulty in determining whether or not sex would occur (for pre-sex dosing) and a mismatch between PrEP dosing and the post-sex milieu	“What would get me to forget is that—I live with my boyfriend, right, okay. So maybe we’re lying on the bed together and then sex just happens… Now my pills sit in a divider and sometimes they are looking at me, but I am busy at the moment… So I will have sex and then will wait for the appropriate time for me to take the after sex pills.” E FG “The regimen that we were in was very difficult. Let’s say that you are in town and your partner phones you and says: “Baby, please come this way when you’re finished in town.” Now you might not have a chance to stop off at home because it could be late.” E FG “And sometimes, after sex, you want to sleep. Maybe you’re tired. You don’t think about taking pills. Maybe you guys are sitting together and talking since you don’t see each other so often. So then you will forget the pills.” E IDI “After sex…. After I have just finished having sex, it’s nice to sit back and relax a bit.” T FG

*D* daily regimen, *T* time-driven regimen, *E* event-driven regimen, *IDI* in depth interview participant, *FG* focus group participant

#### Facilitators of and Challenges to Study Participation

Discourse reflecting reasons for participation, positive or negative consequences of participation, and level of commitment towards and belief in the value of the study and outcomes were reviewed for main themes reflecting facilitators of participation and, conversely, factors challenging participation (Table 3). Participant reflections on facilitators to study engagement were organized into five general themes; (1) Personal experiences with HIV enhancing commitment towards the goals of the study, (2) valuing the package of care received as a participant as unique and beneficial, (3) financial/economic compensation offsetting burden of participation, (4) positive feelings towards the research team, and (5) commitment to HIV prevention research as a benefit to one’s community. Discourse highlighting potential factors negatively impacting full participation or engagement in the study included; (1) Concerns about safety of PrEP and confluence of directly or indirectly experienced side-effects exacerbating these concerns, (2) community distrust of study and/or PrEP and women’s participation in the study expressed as beliefs women were getting treatment for HIV, the clinic was selling blood collected from participants, and devaluation of participants as only interested in money, not community prevention, and (3) negative clinic experiences largely involving discomfort with sexual behaviour questions and a lack of transparency in what was being done with that information, as well as feeling wrongly accused of non-adherence. As indicated in the sample quotes in Table 3, women discussed both sources of pride in being an active participant as well as considerable “cost” in terms of negative pressure from community members and important others. This tension was evidenced across discourse, leading to the further identification of overarching cross-cutting themes that better captured potential drivers, responses and management of these tensions.

**Table 3:** Facilitators of and challenges to study participation

Theme	Defined as discourse on…	Example quotes
Facilitators of study participation		
Personal experiences with HIV	Desires to contribute towards HIV prevention because of negative impact of HIV on family, friends, or community	“I joined because I have a family member who passed on because of HIV, so I decided to take part because I will also benefit”. E FG
Valuing the package of care received as a participant	The unique benefits of being in the study in terms of the medical care and screening not easily available outside of the study	“You know, when we’re in the township, it can be difficult for us to go test at the clinic and you won’t know what your status is. So at least when you come here, you can find out whether you are sick or not. So that supported me because I got to know about my health.” E IDI “Maybe you just want to be cautious about your health because here at the study they look at a lot of things you don’t drink the pills only. That is what I liked” D IDI
Financial/economic compensation	Reimbursements as motivating participation in the study	“They [other participants] also told me about the difficulties they had but then they endured them. Another one told that she is enduring them because you get money in this study, like a lot of money.” T IDI
Positive feelings towards the research team	Experiences, beliefs or feelings towards study team that were positive or motivating	“It’s the way they treat us here at [site name]. It’s the way the counselors also speak to us. They help you understand the way in which these pills are meant to be taken. They don’t force you.” T IDI “All the staff members were supportive I enjoy coming here.” E IDI
Commitment to HIV prevention research	Discourse of a shared vision with the study in terms of working together to make real contributions to HIV prevention in their community	“What made it easy for me was that it’s helping the community. It’s not only helping me. So I am happy that there were people who were supporting me.” D FG “I was following the instructions and I told myself that I was doing it for a purpose. …to check as to whether this research works for other people.” D IDI
Challenges to participation		
Concerns about safety	Study provided PrEP as unsafe or less safe than informed by the research team	“People were not drinking the tablets because they were flushing them down toilets because they were […] experiencing side effects like headaches, stomach ache and gaining weight.” E IDI “…I was scared of getting side effects hence I would throw the pills away….” T IDI “I was okay but got worried because people were talking about side effects.” T IDI
Community distrust of study and/or PrEP and women’s participation in the study	Community rumors/convictions that women would get HIV through participation, have HIV, or prioritize themselves and receiving money for participation over the community	“Yoh! People say that they give you AIDS there!” E FG “…and my friend also said I am looking for trouble by joining this study she had this whole idea of how I could catch HIV.” T FG “And as for my friends… they were telling me that I am only carrying on with this study because I wanted money” D IDI “My family never encouraged me, especially my sister. She just told me that I was going to get AIDS. She said: ‘They take your blood and sell it.’” D FG
Negative clinic experiences	Experiences at clinic site that were negative or considered burdensome; feelings of lack of transparency/feeling accused	“They irritated me because the same question is asked every day: “address, contacts, phone numbers” – all the time… He would ask the same questions. …Then when you come back you have to explain again.” T FG “…and traditionally for us black people we don’t disclose info like that easily to anyone, it’s embarrassing and especially when they ask these unexpected questions.” T FG “…it was all just irritating, and they would look you in the face plus they wouldn’t say if you right or wrong, they would just write down what you saying.” T FG “…the counselors were telling us that we are throwing the pills away, which it was not all of us.” T IDI

*D* daily regimen, *T* time-driven regimen, *E* event-driven regimen, *IDI* in depth interview participant, *FG* focus group participant

#### Cross-Cutting Themes/Narratives Contextualizing Approach to Study-Provided PrEP

Several underlying “cross-cutting” themes were identified. Two reflected cultural contextual factors that likely influenced women’s overall experiences in the study and with PrEP: (1) A prioritization of contributions to the community, which is consistent with a South African worldview *Ubuntu*, and (2) a pervasive skepticism from both participants and communities about the trial, the product, and procedures. The final theme also spanned across discourse but was more reflective of ways in which women used and did not use PrEP: (3) variability in approaches to regimen uptake, persistence and adherence, between participants and over time. Each cross-cutting theme is discussed in detail below, and our integration of these themes into a more comprehensive framework for understanding the experiences of women in the study with hypotheses concerning how factors at the participant, study and community level interacted is provided through a proposed Mutuality Framework.

##### Ubuntu

 Discourse about the personal protective value of PrEP was present but the desire to contribute to something good to the community was resounding. Note that this differed from altruism in that participants reflected on wanting efforts to be valued in *their* community specifically, highlighting strong reciprocity desires. Prioritization of community well-being and feeling aligned with the “good” of one’s society is highly consistent with the concept of Ubuntu. Ubuntu as a worldview emerged in the mid-19th century to describe South African communities working together as communities, and identifies that ‘humanity’ exists in the interactions of groups of individuals. It reflects beliefs that society, over individuals, gives meaning and relevance, emphasizes collective responsibility, and commitment towards health and well-being of one’s community.

##### Skepticism

Narratives reflected multiple experiences that spoke to an underlying skepticism towards study-provided PrEP and research more generally. This could be considered “healthy” skepticism in the sense that the cultural, social and political history in communities participating in this study have very recent and on-going experiences with oppression and discrimination that promote skepticism as an important safety precaution. Ongoing economic disparities and poverty characterize daily life for most participants. Medical establishments and biomedical research centers, even those experienced as providing valuable contributions to the participants, can be affiliated with majority group(s) who are seen as responsible for past injustices and ongoing disparities, or overt negligence towards the safety and rights of members of the community. It is important to note that skepticism as used here is not an outright rejection of the study, but rather an approach to the study that seeks “proof” to build trust or to confirm that the study and products should not be trusted. Importantly, high quality interactions with the study site and staff are not sufficient to overcome negative expectations that are deeply seeded in the history of medical services.

##### Variable Approaches to Study-Provided PrEP

How participants approached study-provided PrEP varied considerably, from active avoidance of taking doses and disclosure of such to the study team, to strong commitments to use PrEP and advocacy in support of the study and PrEP in the community and with other participants (PrEP champions). Several women discussed avoiding PrEP use entirely, and there was ample discourse about “other” participants discarding drug, opening and closing the electronic drug monitoring device (Wisepill™) to appear adherent, and advising other participants to avoid PrEP across study arms. However, there was also considerable discussion about actively engaging other participants and challenging negative stereotypes of participants and skepticism in the community. In between the extremes of intentional avoidance and ‘champions’, women discussed variability in persistence (defined here as periods of consistent engagement with the regimen or commitment to trying to adhere) and execution of regimen (adherence towards a regimen one is trying to take).

### Synthesis of Findings: A Mutuality Framework

**Fig. 1:**
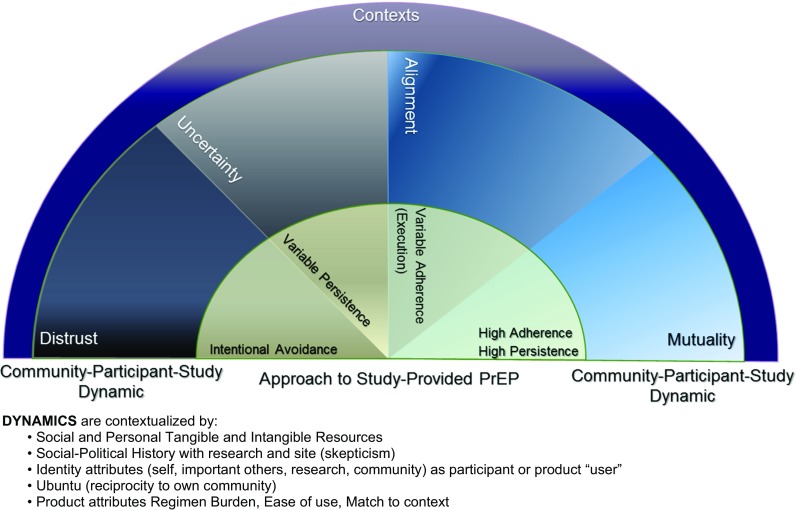
Mutuality framework

The cross-cutting and specific themes were used to develop a framework to understand interactions between participant, community and study and how these influenced women’s approaches to study-provided PrEP. Throughout discourse a tension in negotiating dynamics between self, study and community was clear. We characterize approaches to study-provided PrEP as ranging from intentional avoidance of PrEP dosing to strong persistence and adherence. These approaches are situated within larger social-cultural and resource contexts including; the value of social and personal resources afforded through participation, the social-political community history with biomedical research and medical institutions, identity attributes (how participant and PrEP user is characterized internally, to important others, in the community), cultural world view emphasizing reciprocity to one’s community, and product attributes and regimen burden or ease of use. As indicated in Fig. 1, these “context” factors apply to the formation, maintenance and/or movement between the dynamics.

We adopt the term ‘dynamics’ to refer to the constellation of factors influencing women’s experiences with study-provided PrEP. These are not intended to characterize people, rather they are ways of thinking about the participant’s approach to study-provided PrEP at a given point in time as the result of her negotiation of tensions and synergies between herself, the study and her community. Table 4 presents each dynamic in terms of characterization, approach to study-provided PrEP, drivers of that approach, and implications for intervention. The Mutuality Framework (Fig. 1) and each dynamic detailed below represent our integration of the narratives shared by women in the study, while also expanding beyond the discourse to incorporate findings in previous literature, multiple models and theories pertinent to participatory research, socio-ecological and social-determinants frameworks, and cultural models. As such, the Mutuality Framework we present is advised by factors identified in the current research but extends beyond discourse to propose a new model offering a more complete, contextually-grounded, theoretical conceptualization of experiences with biomedical HIV-prevention.

**Table 4:** Dynamics in Mutuality Framework

Dynamic	Approach to study and study-provided PrEP	Caused by…	Intervention implications and possible strategies
Distrust	Active, intentional avoidance of taking product/PrEP. “…I was scared of getting side effects hence I would throw the pills away….” T IDI “Others were just opening up the container as [proof] that they were taking them while they were not taking them at all.” D IDI	Rejection of integrity of study (goals, potential reciprocity to community) and safety of products/PrEP and efficacy.	Strategies targeting changing beliefs in safety, reciprocity, and efficacy of product or integrity/relevance of research findings (for efficacy trials) Possible change strategies: Community theatre with roles for “pro” and “cons” of drug safety or study integrity where turns are taken in giving voice to each “side”, ending with thoughts on what evidence/experiences would convince one side or the other Normalization of skepticism and overt discussion of pros/cons allowing for exploration of each Community engagement and mobilization events (i.e., CBPR strategies) Designs and programs that allow for discontinuation or not using PrEP while remaining in cohort Creating “task force” teams of participants who are tasked with and resourced to perform fact-finding missions about study and/or products
Uncertainty	Variable persistence with study-provided PrEP- on-again/off-again engagement with trying to use study-provided PrEP. “Firstly, people say that we’re risking our lives by getting involved in HIV research.” E FG “But then I ended up thinking and thinking and thinking about this, whether there really isn’t anything [HIV] they are giving us here.” D IDI	Skeptical exploration of whether or not to trust study, PrEP, or providers of PrEP (the research study, demonstration project, or health agency)	In addition to changing beliefs (above), strategies targeting enhancing beliefs of safety, reciprocity, and efficacy Possible change strategies: PrEP study or program awareness campaigns that invite open discussion of potential medical mistrust from social–historical and political perspectives Promote exploration of ambivalence as reasonable and valid with a focus on identifying what “data” would be needed to assure participant Adopt high transparency strategies that explain aspects of procedures, protocol or PrEP programs that are uncommon in communities- for example, media providing “proof” of legitimacy of tests, samples or monitoring (video of blood collection, where it is shipped with pictures of labs, and disposal after processing) Engage peers, champions, and trusted individuals to lead debates and discussions about integrity, truth, and reciprocity If the PrEP agent is known to be effective, emphasize this aspect of potential value through multiple modalities (pictures, media, theatre and other methods of depicting efficacy) Create opportunities to build sense of ownership in trial- task participants with conducting evaluations of experiences at clinic, quality of care received, and social harms (negative experiences) in community that are fed back to the research team and acted upon
Alignment	Whereas persistence (trying to take study-provided PrEP) is likely good; execution adherence is anticipated to vary on the basis of adherence skills (strategies) and degree of positive beliefs about value of PrEP and adherence “… I wouldn’t do any of that [not take the tablets] because I want to see if these pills really, really work” T FG “I also wanted to continue taking it to the end and if I hear that the pill did its job and helped people, I will be proud of that.” E FG	Provisional acceptance that the study and products provided by study do benefit self and community in ways that are relevant and meaningful	Support should target maintaining trust in study and positive beliefs about study-provided PrEP use and optimizing adherence. Possible change strategies: Barriers based discussions to identify adherence challenges and resources and skills that could be used to address them Peer based support for adherence and developing strategies to promote adherence Real-time monitoring may help to provide reminders and problem solving support as and when needed Exploration of collected dosing data (as available) to identify strengths, barriers and potential strategies
Mutuality	Both persistence and execution adherence are generally high/good “… and I said: “Look here, ask me. And don’t you dare say I have HIV, telling everyone in this shop. We are doing research here… to see whether the pills can protect someone from HIV.” E FG “… [people] in the study must help them. They must be proud to talk about the pills and encourage other people.” T FG	Ownership of PrEP and/or goals of the study or program to the point of advocacy	Support for uptake, persistence and adherence are not generally needed in this dynamic. Rather, avoiding eroding mutuality is essential and developing avenues for collaboration offers opportunities to mobilize participant groups and communities Possible strategies to retain women in this dynamic: Create programs for peer mentors, community champions, and other roles that facilitate advocacy Create sister-groups where women can lead discussions among women in the study or program Create and use rotating participant advisory panels where women can take on valued leadership positions within the study or program Engage women in the development of a plan for how results of the study or program will be disseminated to community and policy makers Facilitate the creation of advocacy groups that can lead local and regional efforts to enhance awareness in communities and represent community with local health ministries, feeding back to community progress and reasons for delays in rolling out diverse prevention strategies

#### Dynamic 1: Distrust

In the distrust dynamic one is anticipated to avoid use of study-provided PrEP because of beliefs that the pills provided are unsafe and there is uncertainty about the protection of participants in the study and limited expectations of community or personal benefit from the perceived high-risk of using study-provided PrEP. The aspect of adherence most impacted in this dynamic is uptake. Normative beliefs center on other participants also avoiding use of the pills, and use of the pills reflecting naiveté in other participants. Women in this dynamic attempt to protect other participants by encouraging non-use and drawing from examples of experiencing side-effects and sero-conversions as proof of conviction that participants are at risk. Efforts from the study team to debunk ‘mis-information’ or rumors that minimize or fail to recognize the legitimacy of concerns are expected to reinforce distrust rather than reduce it. Women in this dynamic are expected to avoid open discussions with the study team about non-use of pills, or appearing non-adherent on self-report or other measures that can be adjusted (e.g., announced pill-counts). Arguably, women experiencing distrust are likely the most difficult to work with from a study team perspective because their lack of trust in the integrity and transparency of the study limit open discourse. They may, however, be identified through a lack of drug concentration in combination with reports of high adherence, which could open a window for discourse. Studies that allow for participation without PrEP use (e.g., a no-PrEP arm) may be better positioned to decrease this dynamic or offer opportunities to women to come off PrEP while they consider or re-consider safety. We hypothesize that the presence of this dynamic is likely in contexts where there are driving structural or economic motivators to participate- strong enough to persuade a woman who is experiencing high levels of distrust and fear associated with taking PrEP to nonetheless enroll and show up for visits and procedures. In contexts where participation in the study does not afford high-value, unique benefits, individuals experiencing distrust would not likely enroll or be retained in the study. Interventions to promote movement out of the distrust dynamic may include community based participatory research practices to reduce some of the factors driving the distrust dynamic, and any strategy that dismantles beliefs about conspiracy, hidden risks, or disregard for safety. Strategies that provide opportunities for participants or patients to take on active roles in monitoring quality of service delivery may similarly work to influence beliefs in the integrity and transparency of programs.

#### Dynamic 2: Uncertainty

Individuals in this dynamic are expected to oscillate between PrEP use and non-use in response to shifts between feeling that PrEP use is safe and accurately represented by the study and feeling that PrEP use is unsafe and that the study mischaracterizes risks involved. The aspect of adherence most influenced in this dynamic is hypothesized to be persistence- as periods of attempting to follow the PrEP regimen is interspersed with periods of avoiding it. Objective measures of drug concentrations may mischaracterize individuals in this dynamic if the window is too short (e.g., dosing in last 3 days or last week) and per-week dosing may be a poor characterization as women would be expected to have some weeks on PrEP and some weeks off of it. Electronic dose monitoring devices may have utility to the extent that they are not overtly manipulated to appear persistent even when not taking PrEP. Women’s experiences in this dynamic are characterized by feeling pulled in different directions and the internal debate over whether or not the pills and the study more generally can be trusted is influenced by ongoing experiences with the pills (e.g., side-effects), study team (e.g., positive and negative experiences with study team members), other participants (e.g., appeals from other participants to trust or reject the study and, relatedly, PrEP), important others (e.g., positive and negative influence of family members and partners), and community (e.g., hearing rumors or being ascribed negative traits [‘selling out’ community for money] or positive ones from others in the community). Normative beliefs of what other participants are doing with their pills are fluid and not crystalized as definitely dumping or definitely taking the pills, while hearing of other women’s experiences is anticipated to be particularly impactful in moving out of this dynamic. We conjecture that the experience of this dynamic is tense and uncomfortable, which resolves only when beliefs shift towards either distrust or stronger alignment with the study. It is unclear how typical education and counseling on adherence may play a part in this dynamic, as it could be argued that clear information and support from the study team could move the participant closer towards the aims of the study. Alternatively, if information appears one-sided (reasons why one should or must use PrEP) or dismissive (stating PrEP is safe without further exploration), it could propel rather than diminish concerns. Adherence counseling focused on identification and remediation of barriers to dosing assumes a shared interest in high adherence, which is likely mismatched for those in the uncertainty dynamic where the participant is still considering her willingness to try a regimen. Like the distrust dynamic, Community Based Participatory Research (CBPR) strategies, engaging individuals in service delivery monitoring, and designs or programs that allow for non-use of PrEP may be helpful. Other strategies that promote discussions of uncertainty and ambiguity focused on decision making around uptake and persistence, preferably with the support of trusted individuals (peers or participant “champions”), may be promising.

#### Dynamic 3: Alignment

Individuals in this dynamic are anticipated to be generally engaged with trying to use PrEP, meaning they are likely persistent but may have challenges to consistent dosing due to commonly reported factors such as mustering motivation to dose in specific situations, remembering, negotiating privacy, or having doses accessible. Pill-use is characterized as persistent but with varying levels of adherence. The study, procedures and the pills are generally seen as safe and the goals of the study are generally considered trustworthy, with potential to benefit one’s self and one’s community. The balance of risk and reward is one of minimal risk and possible benefit. Positive beliefs are strong enough to build resilience to negative community pressure, although actively shifting community beliefs is not a priority. Normative beliefs about what other participants are doing, or not doing, exert less of an impact, and one’s own experiences with PrEP, the study and significant others are more influential. Women in this dynamic attempt to follow recommendations and regimens, and have more resilience in reporting non-adherence back to study team members. A diverse set of strategies may be helpful for women in this dynamic. Strategies using objective markers of PrEP use may assist women in identifying patterns that produce optimal and sub-optimal levels of protection. Of note, the education and counseling offered in many studies and programs that focus on unpacking potential facilitators and barriers to adherence and building skills are likely to be most appropriate for individuals in this dynamic, as open discourse is possible and there is a shared goal of adherence.

#### Dynamic 4: Mutuality

Individuals in this dynamic have a high degree of ownership over PrEP and/or the goals of the study. Persistence and adherence are both anticipated to be high and consistent largely due to strong positive beliefs in PrEP and the study’s ability to make lasting, real contributions to personal and community health and wellness. In this dynamic, women are likely aware of participants in other dynamics (particularly rejection and uncertainty) and community concerns about the study or women participating in it. They appreciate that normative beliefs for PrEP (and biomedical prevention more generally) are diverse and fragmented in the community. Unique to this dynamic is the response women have to these experiences. They respond by overtly challenging the beliefs of others, ‘vouching’ for the integrity of the study and the product(s), and seeking out opportunities in the community to shift beliefs. Their advocacy positions them as “PrEP Champions” in both the community outside of the study and within the study itself. Their accumulated experiences with PrEP can position them as more expert in terms of adherence than the study team, who typically do not use PrEP or have lived experience with taking it. It is not clear that women in this dynamic need study-provided support for persistence or adherence, aside from being responsive to specific questions or issues raised by the participant. It may be more important to avoid the introduction of experiences that may move someone out of the mutuality dynamic, and create new opportunities to allow for thus dynamic in the context of research trials. Asking women in this dynamic to reflect on doses missed (self-reported or objectively monitored) may hold appeal to them if presented as for research or data tracking purposes. However, if framed as for their own benefit, women may feel such conversations with staff or study team members, who themselves have little lived experience with taking PrEP and advocating for it in their communities, belittling or dismissive of their own expertise. Support for adherence may be best positioned as “as needed” for women in this dynamic. Other activities, however, could help to keep women in this dynamic and moreover could engage these women in assisting others. Creating opportunities to serve as peer or participant champions who could support other participants, speak at community events, provide input into policy forums, or advise the study team on recommendations for working effectively with community and participants in other dynamics would likely be more appropriate than a focus exclusively on adherence.

## Discussion

Discourse from predominantly young, unmarried women who participated in the HPTN 067/ADAPT trial suggested that approaches to open-label PrEP provided as daily, twice-weekly plus post-sex dose, or pre- and post-sex dosing varied, although many women spoke of high commitment, persistence and adherence to their regimen. Facilitators and barriers identified to dosing were generally consistent with the literature on adherence to antiretroviral therapy (ART) [30], prevention medications (e.g., hormonal contraception [31]), post-sex dosing challenges [32], as well as recent evaluations of study-product use in FEM-PrEP and VOICE [12–15]. In our sample of women, specific challenges to non-daily dosing appeared centered on the context in which participants had sex (e.g., unplanned, as available and typically outside of one’s home) and the context surrounding post-sex (e.g., where relaxation takes precedence over action-oriented prevention behaviors such as dosing). Moreover, throughout the discourse, women reflected on negotiating the potential use of study-provided PrEP in a context where there were substantial concerns about safety and integrity of the trial and procedures, in many cases not knowing whether or not the PrEP provided could be trusted or the study would indeed benefit one’s community.

From the narratives collected, we constructed a Mutuality Framework which proposes a characterization of how the intersections, or dynamics, between self, community and study impacted overall approaches to study-provided PrEP. We propose diverse sets of strategies that could be implemented by study teams and/or PrEP implementation programs as ways to enhance movement towards alignment. Typical adherence support offered within trials and implementation programs that seek to optimize execution adherence (doses taken as recommended) is well-matched to the alignment dynamic, but may be poorly matched in the remaining three dynamics (distrust, uncertainty, or mutuality). Future research should target the evaluation of CBPR strategies [33] and Good Participatory Practices [34], as well as other innovative approaches, in shifting levels of trust in both biomedical agents and biomedical research. Efforts to engage social behavioural research to measure dynamics that will influence PrEP uptake, through the development of new or adaptation of existing scales (i.e., the Group Based Medical Mistrust Scale [35, 36]), characterising movement through dynamics over time and in response to events and experiences, and strategies to effectively shift distributions in our framework are important next steps.

Although our results are specific to the group of participants engaged in this study and may not characterize experiences of participants not included in the interviews or focus groups, we do believe that the overall framework we developed is generalizable. We cannot, however, presently speak to whether or not the data collected would have differed considerably if the only regimen examined in HPTN 067/ADAPT was a proven one. Having other ‘investigational regimens’, even if the drug itself was open label, may have created challenges to perceived safety and clearly could have challenged feelings of efficacy. Even in light of this, we do believe the Mutuality Framework may apply to other projects and PrEP roll-out in areas where there may be skepticism towards biomedical prevention, PrEP specifically, or the agencies that provide it. Moreover, our framework is highly compatible with models of innovation adoption [37], as well as process models for behaviour adoption [38] and participatory engagement models (cf., [39]), suggesting some applicability to PrEP use more generally. However, future research is needed to evaluate the replication and applicability of the dynamics to “real-world” PrEP use.

The critical role of “medical mistrust” in treatment adherence and research participation is well-established, has clear roots in patterns of discrimination and promotes widespread health disparities [40–43]. Our results add to this literature by offering a nuanced framework for understanding the manner in these factors may play out in biomedical prevention studies. We believe many of the core drivers we identify and manner in which systems interact are generalizable to outside of a research trial. Implications to PrEP roll-out may include careful attention to the distribution of the Mutuality Framework dynamics in targeted communities, and how planned dissemination of PrEP may foster or mitigate skepticism, doubt, and distrust. For example, when PrEP demonstration projects or PrEP-specific clinics offer care beyond that which is available in the community, larger distributions in the distrust and uncertainty dynamics would be expected. Education and awareness activities with trusted sources (peers, community members, traditional healers) in trusted venues (faith-based venues, community organizations) would be expected to speak to those experiencing distrust and uncertainty, while scientific experts at town-halls, speaking events and policy forums may speak more to those in alignment and mutuality dynamics. Promoting and capacitating those in mutuality to lead community campaigns and engage policy-makers directly may have high impact.

 In summary, in combination with results from the main HPTN 067/ADAPT study [16] in Cape Town, women in sub-Saharan Africa found PrEP feasible and promising as a self-directed tool for HIV-prevention. Women approached PrEP in different ways, which we believe was dependent on levels of perceived safety, trust in PrEP and those providing it, and investments in protecting one’s community either from PrEP or with it. Past literature has clearly documented the need for effective, generalizable interventions to promote high levels of collaboration and trust in communities for biomedical intervention, prevention, and treatment [42, 44]. As PrEP implementation programs unfold around the world, there is a real urgency to identify how to “get it right”. As suggested by our results, there are many avenues to consider for how to potentially engage communities around PrEP. Importantly, should our Mutuality Framework offer a replicable, generalizable depiction of experiences with PrEP, there are also avenues that could distance communities and create substantial long-lasting barriers in the adoption of biomedical HIV-prevention innovations. Appreciating the cultural, political and historical factors contextualizing PrEP and other biomedical prevention strategies will be a critical ingredient in successful implementation programs.
